# Supplementary figures and images for: Early control of viral load by favipiravir promotes survival to Ebola virus challenge and prevents cytokine storm in non-human primates
Source: PLoS Negl Trop Dis. 2021 Mar 29;15(3):e0009300. doi: 10.1371/journal.pntd.0009300 (PMC8031739; doi:10.1371/journal.pntd.0009300)

Figure S1

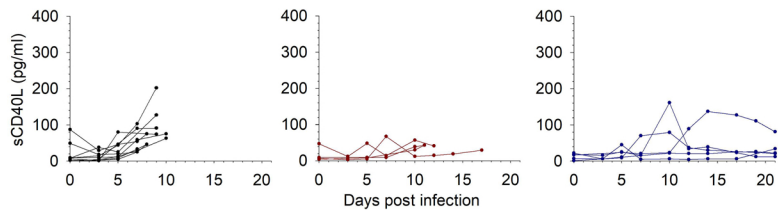

Supplement: S1 Fig — sCD40L was measured at each sampling point during the course of the experiment by Luminex assay. The animals tested and the representation of the values are the same as in Fig 1. (PDF) [file pntd.0009300.s001.pdf]

Figure S3

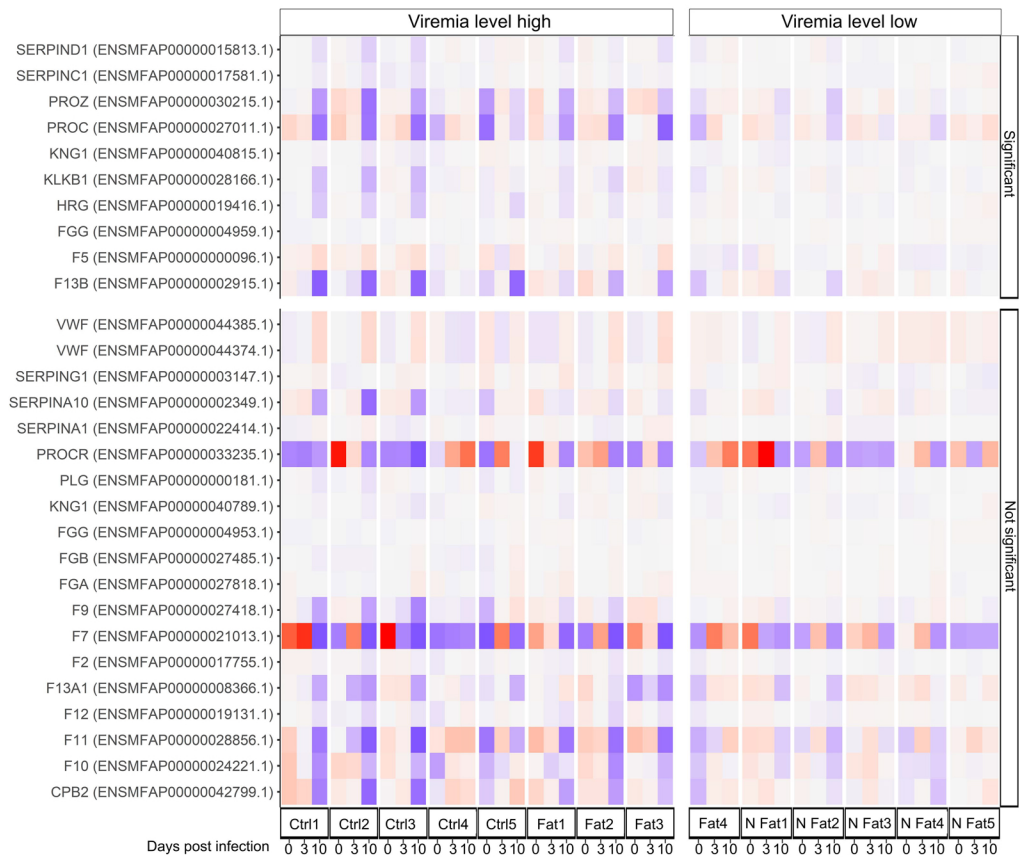

Supplement: S3 Fig — The data is presented as in S2 Fig. (PDF) [file pntd.0009300.s003.pdf]

Figure S4

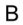

Supplement: S4 Fig — A. Heatmap representing the level of proteins from the complement pathway. B. Representation of the stress-response pathway. The representation and colors for the two heatmaps are as in S2 Fig. (PDF) [file pntd.0009300.s004.pdf]
