# Supplementary material for: Early control of viral load by favipiravir promotes survival to Ebola virus challenge and prevents cytokine storm in non-human primates
Source: PLoS Negl Trop Dis. 2021 Mar 29;15(3):e0009300. doi: 10.1371/journal.pntd.0009300 (PMC8031739; doi:10.1371/journal.pntd.0009300)
Supplement: S2 Fig — Peptides are ranked based on the significance of the regulation of the protein, according to group and the timepoint. The color indicates the evolution of the protein level: red corresponds to upregulation and blue to downregulation. (PDF) [file pntd.0009300.s002.pdf]

Figure S2

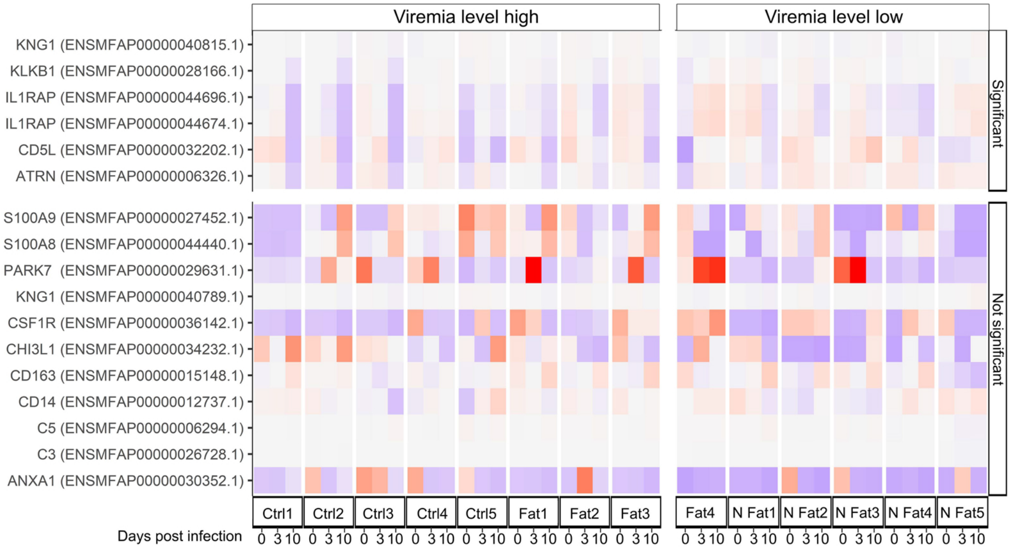
Viremia level high
Viremia level low
KNG1 (ENSMFAP00000040815.1)
KLKB1 (ENSMFAP00000028166.1)
IL1RAP (ENSMFAP00000044696.1)
IL1RAP (ENSMFAP00000044674.1)
CD5L (ENSMFAP00000032202.1)
ATRN (ENSMFAP00000006326.1)
S100A9 (ENSMFAP00000027452.1)
S100A8 (ENSMFAP00000044440.1)
PARK7 (ENSMFAP00000029631.1)
KNG1 (ENSMFAP00000040789.1)
CSF1R (ENSMFAP00000036142.1)
CHI3L1 (ENSMFAP00000034232.1)
CD163 (ENSMFAP00000015148.1)
CD14 (ENSMFAP00000012737.1)
C5 (ENSMFAP00000006294.1)
C3 (ENSMFAP00000026728.1)
ANXA1 (ENSMFAP00000030352.1)
Significant
Not significant
Ctrl1
Ctrl2
Ctrl3
Ctrl4
Ctrl5
Fat1
Fat2
Fat3
Fat4
N Fat1
N Fat2
N Fat3
N Fat4
N Fat5
Days post infection
0 3 10
