# Supplementary material for: Early control of viral load by favipiravir promotes survival to Ebola virus challenge and prevents cytokine storm in non-human primates
Source: PLoS Negl Trop Dis. 2021 Mar 29;15(3):e0009300. doi: 10.1371/journal.pntd.0009300 (PMC8031739; doi:10.1371/journal.pntd.0009300)
Supplement: Attachment — Submitted filename: Rebuttal-PlosNTD.pdf [file pntd.0009300.s005.pdf]

POINT-BY-POINT RESPONSES

We are very grateful for the comments received concerning our manuscript "Early control of viral load by favipiravir promotes survival to Ebola virus challenge and prevents cytokine storm in non-human primates". The concerns made by the reviewer were of great interest and we revised our manuscript to fit with his recommendations. Here, you will find a point by point description of the changes in the manuscript and responses to some of the comments.

- We clarified the description of the statistical analysis performed on proteomic experiments (line 277 – 280). We only compared day 0 between the groups to assess the homogeneity in this time point and we further analyzed the differences in the group to avoid adding a new level of complexity in these experiments that already present numerous statistical values. It seemed to us that the great disparity of the statistical test results between low and high viremia groups are sufficient to justify the differences between the groups for each time point. Moreover, we added the complete protein names in the legend of figures 5 and 6.
- "Why was CD40L not evaluated ?" We actually quantified soluble CD40L. However, we found only low levels of this molecule in control animals and no significant concentrations in treated animals, even those who survived. The expression of this marker was observed in patients who survived in different clinical studies. The levels found in our study were not comparable with the ones measured in humans, we did not consider it necessary to present this data. However, we have discussed this discrepancy (lines 388-396).
- The review from Lancet infectious disease 2011 is considered as a reference and the recent reviews did not explore the coagulation troubles and the impairment of the endothelium. However, we added a more recent publication that corroborates the Lancet from 2011 (line 62).
- "Define G/L." Hematological results were presented in Giga cells / liter (G/L). We defined the unit in the legend (line 152).
- "BUN is referred to as a measurement of kidney function; while this is true, elevated BUN can also reflect dehydration". BUN can actually be associated with dehydration. We modified the text to be less confident in the correlation between BUN levels and renal function (lines 168-169).
- We showed the same figures twice in figures 5C and 6A just to provide all the proteins regulated and associated with the described pathway. These proteins were linked to the two pathways.
- "It is mentioned that one animal did not have detectable viremia." However, viral RNA was detected in this animal and the graph of RNA levels is presented in the same figure. We added the information in the text (line 144).
- Figures were provided to the editor as images in 600 dpi and we checked the quality of each image. If the images were blurry, it is probably due to PDF conversion of the files. However, good quality images are available.

- “The conclusions regarding coagulation are overstated and over simplified.” We deleted some sentences that were too assertive (lines 299, 402 and 408 by extension). The results obtained were not sufficient to explore in details the coagulation cascade and the troubles associated with the dysregulation of these proteins. We thus moderated the statements regarding coagulation dysregulation. Some proteins implicated in the coagulation cascade were not significantly regulated but we looked at precise time points and our statistical method is very stringent, it is possible that we missed some proteins of interest. Factor V and Fibrinogen gamma chain were overexpressed unlike most of the other factors. This is not incoherent with the consumption of coagulation factors as factor V plays a double role of procoagulant and anticoagulant molecule (Duga, IJBCB, 2004) and plasma levels of fibrinogen is related with a poor outcome in ischemic strokes (Bijak, J Cell Mol Med., 2019 and Swarowska, Inflammation, 2014) and thus it could be the same in CIVD.
- The persistence of cytotoxic response in treated animals with no significant difference between fatal and non-fatal cases does not seem to us a contradiction with the lack of efficacy of Favipiravir. Indeed, there is no real dichotomy between the two groups as almost all animals, even those who survived, experienced severe illness. Some of them have been euthanized as they reached the clinical endpoint determined with the ethical committee, but some of them might have eventually survived if no intervention had been done. In accordance with this, we observe different levels of cytotoxic molecules and T cell activation depending on the animal in the different groups. The deleterious role of the immune response in Ebola hemorrhagic fever has been extensively reported; we provide here the evidence that acting on viral load allows to diminish the response linked to CD4 T cells but only partially modulates the cytotoxic response.
- We modulated the conclusions regarding the role of HSP90 in the outcome as this is actually a correlative data (line 416).
- “…Therapeutic interest of the use of antiviral treatments in patients and the interest of acting on inflammation...” The efficiency of drugs and mAbs has always been higher in patients with low viremia. Fatality rate for patients admitted with elevated plasmatic levels of virus remains unacceptable despite the use of the most effective treatments (ref 2, 40, 44). This is the reason why we and others suggest the management of the host response in addition to the antiviral treatment (ref 44) (lines 425-429). In our study, the role of inflammation in the fatality rate is not distinguishable from viral load. However, the treated animals that presented high viral loads had decreased levels of inflammatory molecules and they could survive longer than controls. One of these animals could reach day 19. So, acting on inflammation in addition to the antiviral treatment may benefit to patients.
- Supportive care is commonly used in medical journals to refer to treatments non-specifically directed against the etiological agent but rather intended to act on symptoms. This could include rehydration, antipyretic treatment, immunomodulation, breath assistance, dialysis…
- We have removed the “TM” label contained in the reference #41
