# Supplementary material for: Early control of viral load by favipiravir promotes survival to Ebola virus challenge and prevents cytokine storm in non-human primates
Source: PLoS Negl Trop Dis. 2021 Mar 29;15(3):e0009300. doi: 10.1371/journal.pntd.0009300 (PMC8031739; doi:10.1371/journal.pntd.0009300)
Supplement: Attachment — Submitted filename: Rebuttal_V3-PlosNTD.pdf [file pntd.0009300.s006.pdf]

POINT-BY-POINT RESPONSES

We are very grateful for the new comments received concerning our manuscript "Early control of viral load by favipiravir promotes survival to Ebola virus challenge and prevents cytokine storm in non-human primates". The concerns made by the reviewer were of great interest and we revised our manuscript to fit with his recommendations. Here, you will find a point by point description of the changes in the manuscript and responses to some of the comments.

- "One of the treated animals that was euthanized during the course of the experiment presented injuries. Were they unexplained hemorrhaging, bleeding or bruising ?"
  Precisions of the context of the decision to remove it from the analysis has been added: "because of a wound on the leg that presented signs of necrosis with no evident link with the treatment or the EBOV infection". Lines 79-80.
- "Why have you chosen to study biochemical, virological and hematological parameters ? Is there a direct link to infection ? Or the drug ? Why not other parameters ?
  In this study, we were interested in the improvement of biological parameters depending on the treatment received. We thus analyzed all the parameters known to be dysregulated in EBOV infections and that are of importance in the pathology. We added a sentence in the first paragraph of the results to clarify this point : "To investigate the efficiency of favipiravir antiviral treatment on EBOV infection, the viral loads were measured in plasma samples. Hematological and biochemical parameters were also of interest. Hepatic and renal markers are highly upregulated during EBOV infections together with deep lymphopenia and thrombocytopenia". Lines 144-147.
- In Flow cytometry you were unable to analyze samples from day 3 and changed the protocol? Any reason for that ?
  We clarified this point in the Methods section: "At day 3, we observed that the favipiravir molecule was fluorescent and emitted in the channels associated with the 405 nm laser. Thus, we were unable to analyze these samples and changed the panel of antibodies for the following points to avoid detecting the fluorescence of the treatment". Lines 106-109.
- What is the rationale behind dividing your tested animals into control, treated and fatally-infected and, treated and surviving ?
  We were interested in the parameters that are implicated in the outcome. We thus wanted to compare the measured parameters depending on these criteria and not only the treatment.
- Pictures are blurry and difficult to see; they may consider including tables instead of some graphs.
  We were very careful to provide high definition images for the figures and separated the different groups in different graphs to clarify the view. We think that the files provided to the reviewer were of lower quality than those we provided.
- Can you propose mechanisms of favipiravir MOA or pathogenic events observed during EVD? In other words, is there specific biochemical interaction through which favipiravir produces its pharmacological effect that could be linked to a specific pathogenic event observed during EVD ?
  Favipiravir acts by the inhibition of the viral polymerase and thus decreases the viral loads in infected animals. We clarified this : "Favipiravir is an inhibitor of viral RNA polymerase (27), we thus hypothesize that it decreases the viral load and limits the flood of PAMPs and as a consequence, the intensity of the host response to PAMPs". Line 388-390.
- You mentioned: favipiravir decreases the viral load and limits the fluid of PAMPs, resulting in lower inflammatory responses and reduced pathology. Do you have a supporting evidence from the literature?
  Clinical studies demonstrated that patients who survived from the disease presented lower viral loads and lower levels of inflammatory molecule. This was added in the text with the appropriated

reference: “Favipiravir is an inhibitor of viral RNA polymerase (27), we thus hypothesize that it decreases the viral load and limits the flood of PAMPs, resulting in lower inflammatory responses and reduced pathology as observed in patients that naturally present lower symptoms and recover (28)”. The reference associated corresponds to McElroy, Current Opinion in Virology, 2018. Line 391.

- The control animals synthesized significantly higher levels of chemokines on day 7 than those that were treated, except for IL8. I was expecting to read some discussion about IL8, which was not strongly expressed in most of the controls.

  Statistical analyses were performed at day 7. Unfortunately, we had only one value at day 7 for control animals, we thus were unable to realize the statistical tests for this protein. This corresponds to the following sentence in the text : “Only three of ten animals showed high amounts of this protein, but only one sample was available on day 7 and thus included in the group comparison”. Lines 203-204. We also added a discussion on the variability of cytokine expression in control animals. Lines 367-370.
- I couldn’t see any limitations to their study nor opportunities of future work, it is recommended to conclude the discussion with such remarks.

  These points have been added in the discussion. “Because of evident ethical reasons, the protocols conducted with NHPs comprise few animals and the mortality observed is to be considered with reservation because of the euthanasia of animals with acute syndrome. The results observed in studies such as the one we analyzed here is however very informative in the understanding of the parameters involved in the outcome and in the evaluation of treatment efficiency. Further studies would be needed to improve the dose regimen and the kinetic of treatment (6)”.Lines 434-439
- Find another place for Fig S4, not in the discussion

  This figure that had been added in response to a reviewer’s remark in the previous version has been changed to appear in the results section. Lines 237-241.
- There have been multiple studies looking at EBOV-infection in NHP. I think it would be good for the authors to add some more language to put their findings into context with that already in the literature: what findings reported here are new, which agree or disagree with previous reports.

  Some efforts have already been made to put our study in the context as can be seen with some of the sentences in the discussion: “Previous studies showed that” lines 363-364, “We confirm that” line 368, “Similar results have been reported during severe EVD in humans” line 378, “This discrepancy between humans and NHP could be of interest to investigate” line 413, “It was thus suggested that the inhibition of HSP90 may reduce EBOV replication” lines 430-431.
- In particular, please compare with the results described in the previous paper Madelain et al where some of the parameters were measured. Is this study with different samples or were they processed in a different way ?

  A sentence has been added in the material and methods section to clarify this point: “The data of viral load, IFNα, IL6 and TNFα presented in this article have been previously reported in Madelain et al, 2018 (6), but this study aims at studying the outcome of the disease ; we thus needed to present once more the data depending on our criteria of analysis”. Lines 89-91.
- It would be convenient to state the favipiravir treatment regimen the animals received to save the reader having to go back and look up these references

  This has been modified in the Materials and methods section. Lines 73-74
- “Cytokine expression is directly linked to viremia”, please change to use “correlated”. Line 229.

  The modification has been made. Line 243
- “Cytokine levels are therefore dependent on viral replication”, please change to “correlated. Line 241

  This has been modified. Line 255
- “These results demonstrate that reduction of the viral load by favipiravir treatment resulted in decreased cytokine/chemokine release”, suggest changing to “correlated with decreased cytokine/chemokine release”.Line 363

  This has been modified. Line 379.

- “By acting on viral replication, favipiravir decreases the viral load and limits the flood of PAMPs”, suggest changing to “we hypothesize that…”. Line 372

  This has been modified. Line 394
- Unless I am misunderstanding what it meant here, the sentence starting “Similarly…” seems to actually be opposite of the previous sentence? Please clarify. Line 375

  This sentence was actually poorly worded and was not useful in the paragraph to explain the purpose. We deleted it. Line 398.
- Please change “directly results from” to “correlates with”. Line380

  This has been modified. Line 396.
- “… this improvement”, I think should be read “improving these” Line380

  The sentence has been modified to be more easily understood. Lines 396-397.
- “…the lack of efficacy…”, change to “the incomplete efficacy”. Line 388

  This has been modified. Line 403
- A mAb-based treatment for EBOV has recently been approved by FDA. Please update to reflect this.

  The FDA approval reached on October 2020, during the review process of the paper. We thus added this point in the discussion. Lines 431-432.
- I would take issue with this statement, as the recently FDA-approved mAb therapy did indeed show clinical benefit “in the epidemic setting, when initiated after disease onset”, albeit with incomplete efficacy. Please soften this statement. Line 437

  We really think that the words used “to cure a HIGHLY acute infection such as EVD”, line 455, and “Indeed, patients who came to seek care during the epidemics probably arrived too LATE after symptom onset” lines 440-441 do soften the statement. As described in the references cited that demonstrate this efficacy of the monoclonal antibody cocktail approved by the FDA, the mortality rate was lower in patients treated but the treatment benefited essentially to patients with moderate viral loads. The patients that were admitted for treatment with high viral load did not took so much advantage of the treatment. Lines 442-444.
- I was wondering is this study a part of a clinical trials? Or is there a plan to conduct one ?

  Favipiravir has been tested during EBOV epidemic and the results of this study are cited (references 1 and 2). However, this study was conducted independently from the clinical trial.
